# Thrombophilic Changes and Hematological Complications in Asthmatic Patients with COVID-19: A Systematic Review

**DOI:** 10.3390/diseases13100333

**Published:** 2025-10-08

**Authors:** Gabriela Mara, Gheorghe Nini, Stefan Marian Frent, Ana Lascu, Maria Daniela Mot, Casiana Boru, Coralia Cotoraci

**Affiliations:** 1 Multidisciplinary Doctoral School, Vasile Goldis Western University of Arad, 310414 Arad, Romania; mara.gabriela@uvvg.ro; 2 Faculty of Medicine, Vasile Goldis Western University of Arad, 310414 Arad, Romania; nini.gheorghe@uvvg.ro (G.N.); mot.dana@uvvg.ro (M.D.M.); cotoraci.coralia@uvvg.ro (C.C.); 3 Centre for Research and Innovation in Precision Medicine of Respiratory Diseases, Department of Pulmonology, University of Medicine and Pharmacy Timisoara, 300041 Timisoara, Romania; frentz.stefan@umft.ro; 4 Discipline of Pathophysiology, Department of Functional Sciences, “Victor Babeș” University of Medicine and Pharmacy, 300041 Timișoara, Romania; lascu.ana@umft.ro; 5 Clinic for Cardiovascular Surgery, Institute for Cardiovascular Diseases of Timisoara, 300310 Timişoara, Romania; 6 Centre for Translational Research and Systems Medicine, “Victor Babeș” University of Medicine and Pharmacy, 300041 Timișoara, Romania

**Keywords:** asthma, COVID-19, hematologic complications, D-dimer, thrombosis, venous thromboembolism (VTE), coagulopathy

## Abstract

**Background/Objectives**: The interplay between asthma and COVID-19 raises critical clinical questions, particularly regarding the risk of hematological complications in patients affected by both conditions. While COVID-19 is known to cause coagulopathy and thromboembolic events, it remains unclear whether asthma independently influences these risks. This systematic review aimed to synthesize existing evidence on hematological abnormalities—including D-dimer elevation, thrombocytopenia, and venous thromboembolism (VTE)—in asthmatic patients with confirmed SARS-CoV-2 infection. **Methods:** A systematic search was conducted in PubMed and Web of Science databases for studies published between January 2020 and May 2025. Inclusion criteria were studies reporting hematologic outcomes in asthmatic patients with COVID-19. After duplicate removal, 139 unique articles were screened, with 40 studies meeting inclusion criteria. These included observational cohorts, retrospective analyses, and clinical investigations. Data were synthesized in a systematic review with qualitative synthesis due to heterogeneity in design and reporting. **Results:** The review identified variable patterns of D-dimer elevation and thrombotic events among asthmatic COVID-19 patients. Some studies reported a higher incidence of ICU admission, elevated inflammatory and coagulation markers, and increased thromboembolic risk in asthmatic individuals—particularly those with poor disease control or non-allergic phenotypes. However, findings were inconsistent and often limited by the absence of asthma stratification, standardized outcome measures, and prospective designs. **Conclusions:** Current evidence does not support a definitive link between asthma and increased thrombotic risk in COVID-19. Further research with prospective, phenotype-stratified methodologies and harmonized hematologic endpoints is needed to clarify whether asthma modifies the hematologic trajectory of SARS-CoV-2 infection.

## 1. Introduction

Asthma is a chronic, heterogeneous respiratory disease that affects over 300 million people worldwide and is characterized by persistent airway inflammation, reversible expiratory airflow limitation, and heightened airway responsiveness, resulting in variable respiratory symptoms such as wheezing, shortness of breath, chest tightness, and cough [1] Despite significant advances in research and treatment, both over- and under-diagnosis remain frequent challenges, highlighting the need for continuous updates in evidence-based clinical practice, as reflected in the 2024 Global Initiative for Asthma (GINA) guidelines aimed at optimizing patient-centered care [1]. Asthma involves both innate and adaptive immune activation, leading to airway hyperresponsiveness, epithelial activation, mucus overproduction, and structural remodeling [2]. Asthma pathogenesis involves an imbalance in T helper cell subsets, particularly Th1 and Th2 cells, which inhibit each other’s differentiation [3]. Th1 cells mediate cellular immunity and may suppress asthma development through IgG, IgM, and IgA production, while Th2 cells drive humoral responses, promoting B cell activation, IgE synthesis, and eosinophilic inflammation [4]. Their differentiation from naïve Th0 cells is influenced by the cytokine milieu and dendritic cell (DC) subtypes. Type I DCs, with low IL-12 secretion, fail to induce Th1 polarization, whereas type II DCs promote Th2 differentiation via IL-4. 

The emergence of the coronavirus disease 2019 (COVID-19), caused by the novel severe acute respiratory syndrome coronavirus 2 (SARS-CoV-2), has introduced a new spectrum of clinical challenges across populations with chronic respiratory diseases [5]. A large retrospective study of 39,420 COVID-19 patients found that chronic respiratory diseases—particularly COPD and asthma—were associated with an increased risk of adverse short-term outcomes, but not with higher mortality, while bronchiectasis and chronic respiratory disease (CRD) overlap syndromes showed no significant impact on prognosis [6]. Nevertheless, the interplay between the immunological landscape of asthma and the systemic effects of SARS-CoV-2 infection remains poorly understood.

Importantly, COVID-19 is not confined to the pulmonary system. It is increasingly recognized as a multisystemic disease, exerting substantial effects on the cardiovascular, renal, neurological, and hematological systems [7,8,9,10]. Hematological abnormalities in COVID-19, such as lymphopenia, elevated neutrophil/lymphocyte and platelet/lymphocyte ratios, along with dynamic changes in LDH, CRP, IL-6, procalcitonin, and ferritin, as well as markers of hypercoagulability (e.g., D-dimers, PT/aPTT prolongation, thrombocytopenia), may predict progression to disseminated intravascular coagulation and require early anticoagulation and sustained blood supply management [10,11].

These alterations are largely attributed to a proinflammatory cytokine storm [12], endothelial dysfunction [13], and coagulopathy, constituting what has been termed COVID-19-associated coagulopathy [14,15,16].

Given the inflammatory and immunomodulatory features shared between asthma and COVID-19, an important clinical question arises: *Does asthma modify the risk or nature of hematological complications in SARS-CoV-2 infection?* Patients with asthma may exhibit distinct systemic responses to viral infections, potentially driven by underlying immune dysregulation—particularly in those with a T2-high inflammatory phenotype [17]—and further modulated by long-term controller therapies such as inhaled corticosteroids, leukotriene receptor antagonists, and biologic agents targeting interleukin-5 or immunoglobulin E [18,19].

The aim of this systematic review is to critically evaluate the current evidence regarding hematological complications in asthmatic patients infected with SARS-CoV-2. Specifically, it addresses the prevalence and spectrum of hematologic abnormalities reported in this population, compares the risk and severity of these complications between asthmatic and non-asthmatic individuals, and investigates the potential influence of asthma phenotypes and long-term treatments on hematologic outcomes. By synthesizing available data, this review seeks to elucidate a relatively underexplored interface between viral immunopathology and chronic airway inflammation, with the ultimate goal of informing clinical risk stratification and improving therapeutic approaches for patients affected by both asthma and COVID-19.

## 2. Materials and Methods

This systematic review was conducted and reported in accordance with the Preferred Reporting Items for Systematic Reviews and Meta-Analyses (PRISMA) 2020 guidelines [20]. The review protocol was not prospectively registered in a publicly accessible database (e.g., PROSPERO). 

### 2.1. Search Strategy

A comprehensive literature search was performed in PubMed (*n* = 4042) and Web of Science (*n* = 3487) for articles published between January 2020 and May 2025. While Cochrane and Scopus were not searched, we are confident that the combination of PubMed and Web of Science provided robust and comprehensive coverage of the relevant literature. Search terms included combinations of keywords related to asthma, COVID-19, hematological complications, D-dimer, thrombosis, coagulopathy, and thrombocytopenia. No language restrictions were applied.

### 2.2. Eligibility Criteria

Studies were eligible if they met the following criteria:

- Population: patients with asthma and confirmed SARS-CoV-2 infection.
- Outcomes: hematological complications or laboratory findings, including D-dimer elevation, thrombocytopenia, coagulation abnormalities, and venous thromboembolism (VTE).
- Study design: observational cohort studies, retrospective analyses, case series, and clinical investigations.
- Exclusion criteria were animal studies, in vitro studies, editorials, commentaries, conference abstracts, and single case reports.

### 2.3. Study Selection

**Figure 1:**
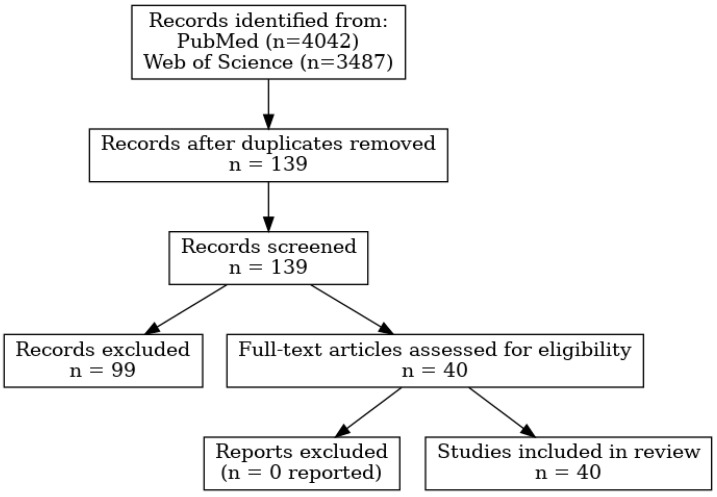
PRISMA 2020 flow diagram summarizing the selection process of studies included in the systematic review.

After removal of duplicates, 139 unique articles were identified. Two independent reviewers screened titles and abstracts, and disagreements were resolved by consensus. Following screening, 99 articles were excluded as irrelevant. A total of 40 full-text articles were assessed for eligibility, all of which were included in the final qualitative synthesis. The study selection process is illustrated in the PRISMA 2020 flow diagram (Figure 1).

### 2.4. Data Extraction and Synthesis

Data extraction was performed independently by two reviewers using a standardized form, and results were cross-checked for accuracy. Extracted data included study design, patient population, asthma phenotype, hematological outcomes, and key findings. Due to substantial heterogeneity in study designs and reporting, a meta-analysis was not feasible; therefore, results were synthesized and grouped thematically (D-dimer, thromboembolism, thrombocytopenia, and coagulopathy). 

## 3. Results

Of the 40 selected studies, 13 cohort and 14 retrospective studies reported data related to D-dimer levels. Most studies observed elevated D-dimer levels in severe cases of COVID-19, regardless of asthma status. Few studies stratified D-dimer levels or thromboembolic events by asthma status. Among those that did, data were not consistent or detailed enough to allow odds ratio or mean difference calculation.

### 3.1. Study Selection

From the 139 unique articles, we identified: 13 cohort studies with relevant data on D-dimers, 13 retrospective studies reporting D-dimer and thromboembolic complications, 9 cohort studies focused on thromboembolism, 4 retrospective studies specifically reporting thromboembolic events. Following the screening and eligibility assessment, 40 studies were included in the final qualitative synthesis. The selection process, including identification, screening, eligibility, and inclusion phases, is summarized in the PRISMA flow diagram (Figure 1).

A total of 7529 records were identified through database searching (PubMed = 4042; Web of Science = 3487). After removing duplicates, 139 unique records remained. Following title/abstract screening, 99 studies were excluded because they did not simultaneously address asthma and hematological outcomes (*n* = 55), reported outcomes unrelated to hematology (*n* = 28), or were non-eligible publication types such as reviews, editorials, or case reports (*n* = 16). The remaining 40 full-text articles were assessed for eligibility, and all were included in the final qualitative synthesis. No additional studies were excluded at the full-text stage.

### 3.2. Study Characteristics

The included studies span a variety of countries (e.g., China, Spain, Italy, USA, Saudi Arabia, Morocco) and cover both adult and pediatric populations. Most cohort studies were hospital-based, while retrospective studies drew on electronic medical records or national databases.

Sample sizes ranged from fewer than 100 to over 10,000 patients. The proportion of asthmatic patients varied from 3% to 20% of each study population.

### 3.3. Hematological Findings

The most common hematological markers evaluated were:

- D-dimer (reported in 27 studies)
- Thromboembolism/VTE (identified in 13 studies)
- Thrombocytopenia and coagulopathy (less frequently reported, but included in ≥10 studies)

D-dimer elevation was significantly associated with hospitalization severity and mortality in asthmatic COVID-19 patients in multiple cohort analyses. Some studies suggested a trend toward higher thrombotic risk in asthmatic patients, while others found no significant difference.

### 3.4. Risk of Bias

Most cohort studies were judged to have moderate-to-high quality, with clear reporting of patient characteristics and hematological endpoints (Table 1). Retrospective studies were limited by potential confounding and inconsistent asthma classification but were still valuable for trend identification.

A comparative synthesis of hematological complications in asthmatic vs. non-asthmatic patients with COVID-19 is summarized in Table 2.

## 4. Discussion

This systematic review with qualitative synthesis synthesized evidence regarding hematological complications in asthmatic patients infected with SARS-CoV-2. While D-dimer elevation and thromboembolic events are well-documented in COVID-19, limited and heterogeneous data exist on how pre-existing asthma modifies these risks.

### 4.1. Hematological Alterations in COVID-19

Severe Acute Respiratory Syndrome Coronavirus 2 (SARS-CoV-2), the causative agent of COVID-19, induces a wide range of hematological abnormalities, varying from subtle changes in blood parameters to life-threatening thrombotic and hemorrhagic complications. These disturbances are the result of a complex interplay between viral infection, systemic inflammation, endothelial dysfunction, and immune activation. Together, they lead to a procoagulant state known as COVID-19-associated coagulopathy (CAC).

#### 4.1.1. D-Dimer Elevation

Elevated D-dimer levels have been consistently reported as one of the most prominent hematological abnormalities in COVID-19 patients [42]. As a fibrin degradation product, D-dimer reflects active coagulation and fibrinolysis, serving as an established indirect marker of thrombotic activity and a key component in the assessment of venous thromboembolism (VTE) risk. In a study involving 97 patients (mean age 63.2 years), elevated admission and peak D-dimer levels—particularly in those requiring intubation (3.79 μg/mL vs. 1.62 μg/mL)—were associated with worse clinical outcomes, suggesting that early monitoring of D-dimer trends may enable timely interventions to reduce the risk of intubation and mortality [43]. Evidence from a retrospective study in two French centers further supports the predictive value of D-dimers: among 10 non-ICU COVID-19 patients receiving thromboprophylaxis, those with deep vein thrombosis (DVT) had significantly higher baseline D-dimer levels (*p* < 0.001). A D-dimer level < 1.0 μg/mL demonstrated a negative predictive value (NPV) of 90% for VTE and 98% for pulmonary embolism (PE), while levels ≥1.0 μg/mL and ≥3.0 μg/mL had positive predictive values (PPV) of 44% and 67%, respectively [44]. In another prospective study of 165 non-ICU patients with COVID-19 pneumonia and D-dimer levels >1000 ng/mL, complete compression Doppler ultrasound identified asymptomatic DVT, underscoring the role of D-dimer in VTE screening in this subgroup [45]. A larger retrospective study of 697 hospitalized COVID-19 patients reported that individuals with pulmonary thromboembolism (PTE)—present in 32.4% of cases—had significantly higher D-dimer levels [median 9.1 μg/mL vs. 2.3 μg/mL; *p* < 0.001]. Although a threshold of 0.3 μg/mL achieved 100% sensitivity, the overall discriminatory performance of D-dimer for PTE was moderate (AUC = 0.77), limiting its reliability as a rule-out test [46]. However, in another cohort of hospitalized patients with clinical signs of severity, a D-dimer threshold > 2590 ng/mL was strongly associated with CTPA-confirmed PE (OR 4.0 per quartile increase; AUC = 0.88), while the absence of anticoagulation therapy further elevated the risk (OR 4.5), emphasizing the importance of D-dimer-guided imaging and systematic thromboprophylaxis in high-risk populations [47]. Finally, a study of 248 COVID-19 inpatients showed that D-dimer levels > 2.14 mg/L at admission predicted in-hospital mortality with 88.2% sensitivity and 71.3% specificity (AUC = 0.85). Moreover, levels > 2.0 mg/L were independently associated with a tenfold increase in mortality risk (OR 10.17, *p* = 0.041), highlighting D-dimer as a robus [48].

SARS-CoV-2 may promote thrombosis through multiple interconnected mechanisms, including cytokine-driven activation of leukocytes, endothelial cells, and platelets, coagulation dysregulation—such as altered PAI-1 and protein C activity—hypoxia-induced vaso-occlusion, and potential direct viral effects on vascular and immune pathways [49,50,51]. However, it remains uncertain whether these thrombotic alterations are specific to SARS-CoV-2 or represent a broader thromboinflammatory response common to viral infections [52].

Autopsy studies have identified neutrophil-rich microthrombi in COVID-19 patients, suggesting a key role for neutrophil extracellular traps (NETs) in the associated thrombotic complications [53,54]. NETs are web-like DNA structures coated with prothrombotic proteins that can activate coagulation via exposure of tissue factor, activation of factor XII, and entrapment of platelets [55,56,57,58]. Similar thrombotic patterns are observed in ARDS, with or without disseminated intravascular coagulation (DIC), where pulmonary thrombi and systemic procoagulant profiles are common. These are characterized by elevated D-dimer and fibrin degradation products (FDPs), decreased protein C levels, and increased concentrations of PAI-1 and soluble thrombomodulin. Enhanced tissue factor expression and impaired fibrinolysis further exacerbate disease severity and contribute to multi-organ failure. Experimental data support this pathophysiological link: neutrophils from hospitalized COVID-19 patients show significantly increased LPS-induced NET formation during the acute phase, and remain primed for NETosis even 3–4 months after infection [59]. Moreover, in a SARS-CoV-2-infected K18-hACE2 mouse model, treatment with DNase I effectively reduced NET accumulation, improved clinical outcomes, and mitigated damage to the lungs, heart, and kidneys—highlighting the therapeutic potential of NET-targeted strategies in severe COVID-19 [60].

#### 4.1.2. Prolonged Prothrombin Time (PT) and Activated Partial Thromboplastin Time (aPTT)

Prolongation of PT and, to a lesser extent, aPTT has been frequently observed in patients with moderate to severe COVID-19. Comparison of 461 COVID-19 and 409 influenza patients (median age 64, 58.7% male) showed that COVID-19 was associated with more pronounced coagulopathy—marked by leukocytosis, neutrophilia, lymphocytopenia, thrombocytopenia, prolonged PT/aPTT, increased INR, reduced fibrinogen, and greater platelet hyperreactivity—while anticoagulated severe COVID-19 cases had a notably higher bleeding risk [61]. In a cohort of 213 confirmed COVID-19 patients admitted in Wuhan between January 31 and 5 February 2020 (median age 62, 44.6% male), the mortality rate was 16.2%, with a median time to death of 6 days. Prolonged prothrombin time (PT), elevated fibrin degradation products, older age, and higher respiratory rate were significant predictors of death. Patients with prolonged PT had significantly lower survival rates (*p* < 0.001), highlighting the prognostic value of coagulation abnormalities in COVID-19 [62]. In a retrospective cohort of 97 COVID-19 patients treated at Dr. Cipto Mangunkusumo Hospital (July–December 2020), 46.4% had poor outcomes. Median coagulation values were PT 11.0” (9.7–28.3), APTT 38.4” (23.9–121), fibrinogen 484.8 mg/dL (51.2–940.9), and D-dimer 1800 µg/L (190–35,200). Poor outcome patients had significantly longer PT and APTT and higher D-dimer levels (*p* < 0.05), while fibrinogen levels were lower but not statistically significant (*p* > 0.05). The results underscore the association of altered coagulation parameters—particularly PT, APTT, and D-dimer—with COVID-19 severity [63]. While these changes may resemble disseminated intravascular coagulation (DIC) in COVID-19 patients. In a multicenter cohort of 23,054 COVID-19 patients, DIC was present in 1.1% on admission and increased to 10.9% by day 15. Early DIC was moderately predictive of MODS and in-hospital mortality, while DIC diagnosed between days 8 and 15 was strongly associated with reduced survival and higher death risk [64].

#### 4.1.3. Thrombocytopenia

It is known that a mild thrombocytopenia state can exist in approximately 70–95% of COVID-19 patients [65]. In a cross-sectional study of 117 COVID-19 patients in Northwest Ethiopia (mean age 50.6 years, 65.8% male), thrombocytopenia was observed in 23.9% of cases. Compared to mild cases, thrombocytopenia was 4.57 times more likely in moderate cases and 6.10 times more likely in severe cases. Platelet distribution width (PDW) was also significantly associated with disease severity (*p* = 0.001), suggesting that platelet indices, particularly thrombocytopenia and PDW, may serve as markers of COVID-19 progression [66]. Xu et al. proposed three main mechanisms for COVID-19-associated thrombocytopenia: (1) reduced platelet production due to bone marrow suppression by viral invasion and cytokine-induced damage to hematopoietic progenitors; (2) increased platelet destruction through immune-mediated mechanisms involving autoantibodies and immune complexes; and (3) increased platelet consumption caused by lung injury and microthrombi formation in the pulmonary circulation [67].

#### 4.1.4. Venous Thromboembolism (VTE)

COVID-19 is now recognized as a highly prothrombotic disease. In a large retrospective cohort of 398,530 nonhospitalized COVID-19 patients in California, the overall venous thromboembolism (VTE) rate was low (0.26 per 100 person-years), with the highest risk observed within the first 30 days post-diagnosis (0.58 per 100 person-years). Independent predictors of increased VTE risk included older age (HR up to 6.51 for ≥85 years), male sex (HR 1.49), prior VTE (HR 7.49), thrombophilia, inflammatory bowel disease, and higher BMI (HR 3.07 for BMI ≥ 40) [68]. The incidence of venous thromboembolism (VTE), including deep vein thrombosis (DVT) and pulmonary embolism (PE), is significantly higher among hospitalized and especially ICU patients. A meta-analysis of 42 studies involving 8,271 COVID-19 patients found an overall venous thromboembolism (VTE) rate of 21%, rising to 31% in ICU patients. Deep vein thrombosis occurred in 20% (ICU: 28%), and pulmonary embolism in 13% (ICU: 19%). Arterial TE was less common (2% overall; ICU: 5%). TE was associated with significantly higher mortality (23% vs. 13%), with a pooled odds ratio for death of 1.74 (95% CI: 1.01–2.98; *p* = 0.04), indicating a 74% increased mortality risk in patients with TE [69].

### 4.2. The Role of Asthma in Coagulopathy and Thrombosis

Despite the chronic inflammatory nature of asthma, the interaction between asthma and SARS-CoV-2-induced coagulopathy remains poorly delineated. A retrospective single-center cohort study compared 21 asthmatic COVID-19 patients with 100 matched non-asthmatic controls based on age, sex, and comorbidities. Asthmatic patients showed higher ICU admission rates and elevated levels of inflammatory markers such as IL-6, IL-8, and procalcitonin. Notably, they also exhibited significantly increased D-dimer levels, indicating heightened activation of coagulation pathways. These findings suggest that individuals with asthma may have an amplified thrombo-inflammatory response when infected with SARS-CoV-2, potentially predisposing them to a greater risk of thromboembolic complications [29]. Several cohort and retrospective studies reported elevated D-dimer levels in COVID-19 patients with asthma, but did not stratify data sufficiently to allow formal comparison with non-asthmatic patients. Some studies observed numerically higher thromboembolic events in asthmatic individuals, yet lacked statistical power or proper subgroup analysis.

A multicenter retrospective study conducted across 13 allergy departments in Spain evaluated 201 asthmatic patients diagnosed with COVID-19. Approximately 30% required hospitalization for bilateral pneumonia. Severe COVID-19 was associated with older age, cardiovascular comorbidities, eosinopenia, lymphopenia, and elevated D-dimer levels—an established marker of thromboembolic risk. Conversely, patients with allergic or eosinophilic asthma phenotypes, characterized by elevated IgE levels, aeroallergen sensitizations, and blood eosinophilia, exhibited milder disease courses and reduced hospitalization rates. Notably, poor asthma control and reduced FEV1 prior to infection correlated with worse COVID-19 outcomes. These findings suggest that specific asthma phenotypes may influence the inflammatory-thrombotic axis differently, potentially modulating the risk of SARS-CoV-2-related coagulopathy [30].

This inconsistency is due in part to methodological limitations, such as lack of asthma phenotype classification, variation in D-dimer assay thresholds, and the use of retrospective designs.

### 4.3. Potential Explanatory Mechanisms

Several hypotheses may explain the unclear role of asthma in modulating hematologic outcomes during COVID-19. Inhaled corticosteroids (ICS), frequently prescribed in asthma, may exert protective anti-inflammatory and anticoagulant effects. ICS are known to downregulate ACE2 and TMPRSS2 expression in airway epithelium [70], potentially limiting viral entry and subsequent systemic inflammation. Conversely, severe or eosinophilic asthma phenotypes may potentiate inflammatory responses that amplify the risk of coagulopathy [71]. Eosinophils release tissue factor and interact with platelets, contributing to thrombus formation [72,73]. Comorbidities frequently co-existing with asthma (e.g., obesity, hypertension) may independently influence coagulation pathways and confound observed associations [74,75].

Asthma is a heterogeneous disease, and its phenotypes may differentially influence the risk of hematological complications in the context of COVID-19. Type 2 (T2)-high asthma, characterized by eosinophilic inflammation and elevated IL-4, IL-5, and IL-13 pathways, has been associated with a more robust Th2 response, which may mitigate the hyperinflammatory state triggered by SARS-CoV-2 infection [76,77]. Conversely, patients with T2-low asthma, who often display neutrophilic or paucigranulocytic inflammation, may not benefit from such immunomodulation and could therefore be more vulnerable to systemic inflammation and coagulation abnormalities [78].

Inhaled corticosteroid (ICS) therapy represents another important factor that may influence outcomes. ICS reduce airway inflammation and downregulate ACE2 and TMPRSS2 expression in the respiratory epithelium, potentially decreasing viral entry and replication. Several observational studies have suggested that regular ICS use is not associated with increased COVID-19 severity and may even exert a protective effect by limiting the progression toward systemic hyperinflammation, which in turn reduces the risk of hematological complications such as elevated D-dimer levels, coagulopathy, and venous thromboembolism. However, evidence remains heterogeneous, and most studies have not stratified outcomes according to asthma phenotype and ICS treatment simultaneously [79]. Further prospective investigations are needed to clarify whether the interaction between asthma endotypes (T2-high vs. T2-low) and ICS therapy modulates the hematological risk profile in COVID-19 patients.

**Figure 2:**
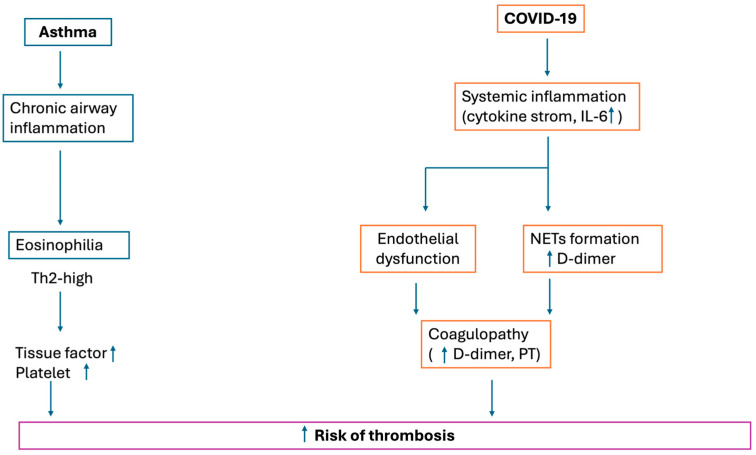
Conceptual model illustrating shared and distinct pathophysiological pathways linking asthma and COVID-19 to hematologic complications, including hypercoagulability and thrombotic risk.

To better illustrate the potential mechanisms linking asthma, SARS-CoV-2 infection, and hematologic complications, we propose a conceptual model integrating the key pathophysiological pathways involved (Figure 2).

### 4.4. Gaps in the Literature

This review identified several recurring limitations:

- scarcity of studies that stratify hematologic data (D-dimer, VTE, platelets) by asthma status;
- lack of standardized outcome reporting for hematologic parameters;
- heterogeneity in asthma definition and severity assessment across studies;
- absence of prospective studies assessing dynamic hematological changes in asthmatic COVID-19 patients.

Moreover, the overrepresentation of hospitalized and severe cases introduces potential selection bias, limiting generalizability to mild or community-managed COVID-19.

### 4.5. Clinical and Research Implications

In the clinical management of patients with concomitant asthma and COVID-19, a vigilant approach toward potential hematologic complications is warranted, particularly in the context of coexisting risk factors. Nonetheless, in the absence of robust, phenotype-specific evidence, asthma per se should not be assumed to independently alter thrombotic risk.

To advance understanding in this area, future investigations should adopt prospective study designs incorporating longitudinal follow-up; stratify cohorts according to asthma phenotype, level of disease control, and pharmacologic treatment regimens; employ harmonized and clinically relevant definitions of hematologic outcomes; identify and validate biomarkers predictive of hematologic risk within this specific clinical subset.

### 4.6. Clinical Monitoring and Anticoagulation

Available data do not justify tailoring hematologic monitoring or anticoagulation strategies on the basis of asthma alone in patients with COVID-19. Thromboprophylaxis and treatment decisions should remain guided by established, risk-based indicators (disease severity, immobilization, prior VTE, obesity, rising D-dimer, and bleeding risk). Routine monitoring (complete blood count, coagulation parameters, and D-dimer when clinically indicated) should follow standard institutional or guideline-based pathways. Asthma-related factors may warrant heightened vigilance—not protocol changes—when additional risks coexist, such as poor asthma control or T2-low/neutrophilic phenotypes, obesity, advanced age, or recent/systemic corticosteroid use. At present, there is no evidence to support escalation of anticoagulation intensity solely due to asthma in COVID-19. Prospective, phenotype-stratified studies are needed to determine whether specific asthma endotypes or therapies (e.g., inhaled corticosteroids) meaningfully modify thrombotic risk [80].

It should be noted that anticoagulation with LMWH was inconsistently reported across the included studies. In hospitalized cohorts, prophylactic LMWH was frequently administered according to institutional guidelines, but many reports did not specify thromboprophylaxis status. This limitation precludes firm conclusions on the role of anticoagulation in modulating thrombotic risk in asthmatic patients with COVID-19.

### 4.7. Limitations

This systematic review has several limitations. First, the included studies were highly heterogeneous in terms of design, patient population, and reporting of hematological outcomes, which precluded quantitative pooling of data. Second, most studies were observational and retrospective, limiting causal inference. Third, information on important clinical factors, such as asthma phenotype (T2-high vs. T2-low), disease control, comorbidities, and use of inhaled corticosteroids, was often incomplete. Fourth, data on thromboprophylaxis and anticoagulation were inconsistently reported, preventing systematic analysis of their impact on thromboembolic risk. 

### 4.8. Future Directions

Future research should focus on prospective, multicenter studies with standardized reporting of hematological parameters in asthmatic versus non-asthmatic patients with COVID-19. Stratification by asthma phenotype (T2-high vs. T2-low) and by treatment (particularly inhaled corticosteroids and biologics) is needed to clarify their role in modulating thrombotic risk. Moreover, better reporting of anticoagulation practices and outcomes will help establish whether asthma modifies the effectiveness of thromboprophylaxis. Finally, long-term follow-up studies are required to assess whether hematological complications persist beyond the acute phase of infection in asthmatic populations.

## 5. Conclusions

This systematic review underscores the limited but emerging evidence on hematological complications in asthmatic patients with COVID-19. While D-dimer elevation and thromboembolism are frequently reported in COVID-19, the current literature lacks sufficient data to evaluate the independent impact of asthma. Future studies should focus on asthma phenotype, treatment, and standardized hematological reporting.

## Figures and Tables

**Table 1:** Characteristics of the studies included in this systematic review.

Authors and Year of Publication	Methodology	Objective	Results
Park et al., 2022 [21]	Cohort study	To evaluate the risk of new-onset respiratory conditions, including asthma, after COVID-19 hospitalization	SARS-CoV-2 infection increased the 60-day risk of asthma (aOR = 1.37) in previously unaffected patients
Greuel et al., 2021 [22]	Retrospective autopsy case series	To investigate clinical histories, comorbidities, and autopsy findings in COVID-19 decedents aged ≤50 years.	Asthma was a common comorbidity; thromboembolism, coagulopathy, and immune dysregulation were frequent findings at autopsy.
Whittaker et al., 2021 [23]	Population-based retrospective cohort study	To analyze GP consultation rates for post-COVID-19 conditions, including asthma, in hospitalized and community-managed patients.	Asthma-related consultations decreased after the first COVID-19 vaccine dose among community patients. No increase in asthma incidence post-COVID was highlighted.
Whichmann et al., 2021 [24]	Prospective autopsy-based cohort study	To compare clinical and virological data with findings from complete medical and virtual autopsies in early COVID-19 deaths.	Asthma or COPD was present in 25% of cases. Deep vein thrombosis was found in 58% of patients, with pulmonary embolism as the direct cause of death in 33%. Findings support COVID-19-related coagulopathy as a key contributor to mortality.
Abunasser et al., 2023 [25]	Retrospective follow-up cohort study	To describe characteristics, outcomes, and mortality predictors in COVID-19 ARDS patients requiring tracheostomy.	Asthma was a common comorbidity. Venous thromboembolism occurred frequently. Asthma, COPD, and renal replacement therapy were associated with prolonged ventilator dependence. One-year survival was 55.5%.
Ramos-Martinez et al., 2021 [26]	Observational study	To determine readmission rates, causes, and associated risk factors after COVID-19 hospitalization	Asthma (OR = 1.52) was an independent risk factor for 30-day hospital readmission. Venous thromboembolism was among the main causes of readmission (5%).
Tseng et al., 2025 [27]	Retrospective cohort study	To compare circulatory and respiratory sequelae after SARS-CoV-2 reinfection vs. initial infection, and assess the role of vaccination.	Reinfection was associated with reduced risk of asthma (HR = 0.791) and venous thromboembolism (HR = 0.741) compared to initial infection. Vaccination offered protective effects against severe circulatory and respiratory complications.
Elmelhat et al., 2020 [28]	Retrospective observational study	To compare the effects of prophylactic vs. therapeutic doses of enoxaparin in severe COVID-19 cases.	Bronchial asthma was present in 1.7% of patients. Venous thromboembolism risk was not significantly different between groups. Prophylactic dosing was associated with shorter ICU stay and less need for mechanical ventilation.
Jin et al., 2022 [29]	Single-center retrospective cohort study	To analyze clinical and laboratory differences between COVID-19 patients with and without asthma	Asthma group showed higher ICU admission rate (14.3% vs. 2.1%, *p* = 0.040). Inflammatory and hematologic markers were significantly elevated: D-dimer, LDH, hs-cTnI, IL-6, IL-8, neutrophils, CD4+ T cells. Results suggest heightened inflammation and multiorgan damage in asthmatic COVID-19 patients.
Habernau Mena et al., 2022 [30]	Multicenter retrospective cohort study	To assess COVID-19 severity in asthmatic adults and its association with phenotype, biomarkers, and lung function.	Among 201 asthmatic COVID-19 patients, ~30% were hospitalized for bilateral pneumonia. Severe cases were associated with older age, high D-dimer, eosinopenia, and heart disease. Allergic/eosinophilic asthma phenotypes showed better outcomes and fewer admissions.
Abdelghany et al., 2022 [31]	Single-center retrospective cohort study	To determine the prevalence and prognostic impact of chronic respiratory diseases, including asthma, in hospitalized COVID-19 patients.	CRDs were present in 17.6% of patients. Asthma included among comorbidities. D-dimer and LDH were more elevated in non-CRD patients. No significant difference in mortality, but CRD patients were more hypoxemic and more frequently discharged with home oxygen.
Al-Ghamdi et al., 2022 [32]	Retrospective cohort study	To assess survival and mortality predictors in hospitalized COVID-19 patients in Saudi Arabia.	Asthma and older age were associated with increased mortality. High D-dimer levels were marginally predictive (*p* = 0.05). Heart failure and renal failure were the strongest predictors of death.
Bonifazi et al., 2021 [33]	Multicenter retrospective cohort study	To assess prognostic predictors, including asthma and D-dimer levels, in hospitalized COVID-19 patients aged ≤50 years.	Asthma and elevated D-dimer were independently associated with increased in-hospital mortality. Obesity predicted mechanical ventilation but not death. Findings highlight the prognostic relevance of asthma even in younger patients.
Hodes et al., 2022 [34]	Retrospective comparative cohort study	to assess and compare the rate of pulmonary embolism (PE) in pediatric versus adult patients with acute COVID-19 using CTPA.	PE was present in 14% of pediatric and 18% of adult cases. All pediatric PE cases were >18y, obese, and had asthma or other comorbidities. No PE was observed in children <18y. In adults, elevated D-dimer levels were significantly associated with PE (*p* = 0.004).
Brito et al., 2024 [35]	Prospective cohort study	To describe clinical-laboratory profiles and identify predictors of COVID-19 severity	Asthma significantly increased the risk of severe disease (OR = 4.58). Elevated D-dimer (OR = 1.26) and platelets were also associated with severity. Other predictors included liver enzymes and electrolyte disturbances.
Mana et al., 2020 [36]	Multicenter retrospective case series	To describe the clinical characteristics of non-intubated COVID-19 patients who developed spontaneous subcutaneous emphysema and pneumomediastinum	Among 11 non-intubated patients with SE/SPM, 27% (3/11) had asthma. Median time to SE onset was 13.3 days. D-dimer and IL-6 were elevated. Mortality was 36%. SE/SPM occurred without mechanical ventilation, suggesting other COVID-specific mechanisms.
Pandey et al., 2023 [37]	Prospective cohort study	To evaluate hematological and clinicoradiological prognostic markers in young COVID-19 patients during the second wave	Asthma was associated with increased mortality (1.1% of total cases). Non-survivors had higher D-dimer levels (mean = 74.87 ng/mL), CRP, LDH, and neutrophils. CT score >15 and mechanical ventilation were strong mortality predictors
Baba et al., 2022 [38]	Retrospective observational cross-sectional study	To assess the diagnostic performance of the WELLS score and D-dimer levels for pulmonary embolism in COVID-19 patients	PE was confirmed in 47/77 patients (61%). Asthma was present in 5.26% of PE cases. Elevated D-dimers and comorbidities were frequent among deceased patients (38.3% mortality in PE group). WELLS score combined with D-dimer improved diagnostic accuracy
Adrish et al., 2020 [39]	Single-center retrospective cohort study	To evaluate the impact of ACE/ARB therapy on outcomes in hospitalized COVID-19 patients, including those with asthma	Among 469 patients, 19.4% received ACE/ARB. Asthma was more frequent in this group. No significant difference in D-dimer levels between groups. ACE/ARB use was associated with higher survival (*p* = 0.0062)
Elesdoudy et al., 2022 [40]	Retrospective analytical cohort study	To assess endocrine and hepatic dysfunction in severe and critically ill COVID-19 patients	Among 75 ICU/HDU patients, 5.3% had asthma. Significant changes in glucose, thyroid hormones (TSH, fT4), liver enzymes, and electrolytes were observed during hospitalization. These parameters may serve as surrogate biomarkers for disease progression
Elesdoudy et al., 2022 [41]	Retrospective observational study	To assess the efficacy of ivermectin in treating severe and critically ill COVID-19 pneumonia patients	Among 50 ICU/HDU patients, 6% had asthma. Clinical and radiological status deteriorated in 36%, improved in 16%, and remained unchanged in 48%. No significant difference was found in inflammatory or hematologic markers before and after ivermectin use. The treatment was not associated with clinical or laboratory improvement

**Table 2:** Comparison of hematological outcomes in asthmatic vs. non-asthmatic COVID-19 patients.

Hematological Outcome	Asthmatic COVID-19 Patients	Non-Asthmatic COVID-19 Patients	Comparative Interpretation
D-dimer elevation	Frequently reported, but usually not markedly higher compared with controls; associated with moderate disease severity	Strongly elevated in severe and critical COVID-19; consistently linked with ICU admission and mortality	Asthmatic patients show milder D-dimer elevations
Thrombocytopenia	Rare; most studies report normal or only slightly decreased platelet counts	More common in severe cases; associated with poor outcomes	Thrombocytopenia appears less prevalent in asthmatics
Coagulopathy	Limited cases; inconsistent abnormal coagulation parameters	Frequent in severe disease; prolonged PT/aPTT, elevated fibrinogen degradation products	Evidence suggests lower coagulopathy burden in asthmatics
Venous thromboembolism (VTE)	Reported but less frequent; few cohort studies confirm events	Common in hospitalized/severe patients; major cause of morbidity and mortality	Asthma does not appear to significantly increase VTE risk; incidence is lower in asthmatics

Note: Findings are synthesized from observational studies; heterogeneity between study designs precluded quantitative pooling.

## Data Availability

Not applicable.
